# Expanding the Phenotypic Spectrum of NDUFS6-Related Disease: From Neonatal Mitochondrial Encephalopathy to Childhood-Onset Axonal Neuropathy

**DOI:** 10.3390/ijms27031375

**Published:** 2026-01-29

**Authors:** Savas Baris, Rojan Ipek, Saniye Tugba Baris, Ibrahim Baris

**Affiliations:** 1 Department of Medical Genetics, Aydın Maternity and Children’s Hospital, Aydin 09020, Türkiye; 2 Department of Pediatric Neurology, Dicle University, Diyarbakır 21280, Türkiye; 3 Gelisim Medical Laboratories, Division of Genetics, Istanbul 34100, Türkiye; 4 Department of Molecular Biology and Genetics, Koc University, Istanbul 34450, Türkiye

**Keywords:** NDUFS6, epilepsy, CMT, neuropathy

## Abstract

Biallelic variants in *NDUFS6*, encoding an accessory subunit of mitochondrial complex I, were initially associated with lethal neonatal mitochondrial encephalopathy and Leigh syndrome. Recent studies have demonstrated that *NDUFS6* variants can also cause childhood- or adolescent-onset axonal neuropathy and Charcot–Marie–Tooth (CMT)-like phenotypes, indicating marked clinical heterogeneity. Here, we report a patient with a novel homozygous truncating *NDUFS6* variant presenting with a neuropathy-predominant phenotype accompanied by epilepsy, in the absence of neonatal metabolic decompensation. The patient presented with childhood-onset progressive gait abnormality, pes cavus deformity, distal weakness requiring Achilles tendon-release surgery, pyramidal signs, urinary incontinence, and focal epileptiform EEG findings. Brain MRI showed bilateral lenticular nucleus abnormalities. Whole-exome sequencing identified a novel homozygous *NDUFS6* nonsense variant (c.130C>T, p.Gln44*). While neuropathy has previously been reported primarily in association with the recurrent splice-site variant c.309+5G>A, our findings demonstrate that truncating *NDUFS6* mutations can also underlie a neuropathy-predominant phenotype. Together with previously published cases, our findings support a phenotypic heterogeneity ranging from lethal encephalopathy to neuropathy and reinforce the role of *NDUFS6* as a disease-causing gene for inherited peripheral neuropathy. These data support inclusion of *NDUFS6* among established neuropathy and Charcot–Marie–Tooth genes.

## 1. Introduction

*NDUFS6* is a nuclear-encoded mitochondrial protein that localizes to the matrix arm of respiratory chain complex I (CI). Complex I, also known as NADH:ubiquinone oxidoreductase, EC 1.6.5.3, is the largest and most intricate enzyme of the oxidative phosphorylation (OXPHOS) system and plays a central role in cellular energy metabolism. Composed of 45 subunits, including 14 catalytic core subunits and multiple accessory subunits, complex I is essential not only for electron transfer but also for maintaining mitochondrial structural stability and assembly integrity [1,2]. Clinically, complex I deficiency is associated with Leigh syndrome; mitochondrial encephalomyopathy, lactic acidosis, and stroke-like episodes (MELAS); Leber hereditary optic neuropathy (LHON); and cardiomyopathies. Patients showed marked phenotypic heterogeneity, with presentations ranging from fatal infantile lactic acidosis to more moderate phenotypes, including spastic quadriplegia with lactic acidosis, cardiomyopathy with cataracts, hepatopathy, and renal tubulopathy, as well as milder manifestations accompanied by chronic lactic acidemia [3,4].

Peripheral neurons are particularly vulnerable to complex I dysfunction because of their high energetic demands, long axonal architecture, and reliance on efficient mitochondrial transport and ATP production. Consequently, defects in nuclear-encoded complex I subunits—including NDUFS2 (OMIM 602985), NDUFS6 (OMIM 603848), NDUFS7 (OMIM 601825), NDUFV2 (OMIM 600532), and NDUFA1 (OMIM 300078)—have increasingly been recognized as an important cause of inherited neuropathy and Charcot–Marie–Tooth (CMT)-like disease [5,6,7].

*NDUFS6* encodes a nuclear-encoded accessory subunit located in the matrix arm of complex I and contributes to stabilization of the N-module [8]. Mutations in the *NDUFS6* gene have been primarily associated with severe disease, most commonly reported as lethal infantile mitochondrial disease (LMID) or Leigh syndrome (LS), with onset typically before 6 months of age and a rapidly progressive clinical course. In early reports, affected patients died within the first days to weeks of life, due to central hypoventilation or refractory lactic acidosis despite treatment [9,10,11,12,13,14,15].

Initial reports of *NDUFS6* pathogenic variants described neonates with severe lactic acidosis, respiratory failure, and early death, implying the gene as a cause of lethal infantile mitochondrial disease. However, recent reports have suggested that *NDUFS6*-related disease may not be restricted to neonatal encephalopathy. A childhood-onset axonal neuropathy with optic atrophy was reported in a male patient with homozygous c.309+5G>A mutation demonstrating prolonged survival and predominant peripheral nervous system involvement [7]. Additionally, Armirola-Ricaurte et al. [16] identified multiple families with the same c.309+5G>A homozygous *NDUFS6* splice variants presenting with axonal Charcot–Marie–Tooth (CMT) disease, pes cavus deformity, and slow progression, often without significant metabolic derangement.

These observations indicate that NDUFS6 deficiency encompasses a broader phenotypic spectrum than previously appreciated. Here, we report a new patient with a novel homozygous truncating *NDUFS6* variant and neuropathy-dominant features and present a review of all published cases to refine the clinical and phenotypic boundaries of *NDUFS6*-related disease.

## 2. Results

The patient was a 13-year-old girl referred for evaluation of progressive gait abnormalities. The patient was born by the non-cesarean vaginal route. Early neurodevelopment was normal until three years of age, when toe walking and gait instability became apparent. At six years of age, she underwent Achilles tendon-release surgery due to severe distal contractures. Additional history included urinary incontinence. Distant parental consanguinity was present. Family history was notable for epilepsy in cousins, frequent falls and persistent W-sitting in a sibling, and exertional leg pain and early fatigue in the mother.

**Figure 1:**
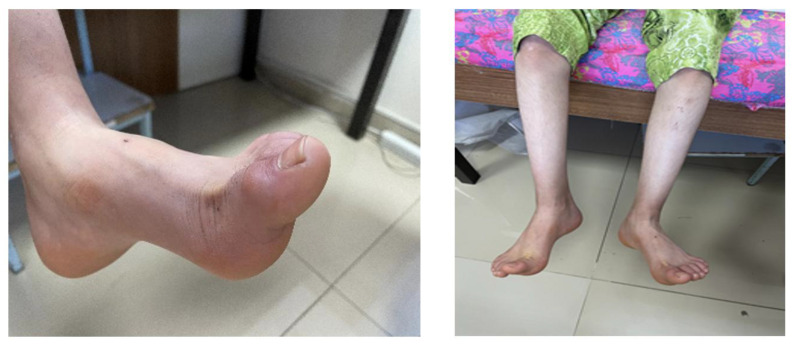
Bilateral pes cavus deformity in patient.

At physical examination, the patient was mentally well. Muscle strength was moderately reduced (3–5/5). Mild scoliosis was observed. Deep tendon reflexes were increased, more prominently in the lower extremities. Gait examination revealed a knee-flexion gait with impaired heel and tandem walking, consistent with distal motor involvement and pyramidal tract signs. Knee-fracture gait was present, heel and tandem gait was impaired, and pes cavus deformity and a surgical scar on the right Achilles tendon were observed (Figure 1).

**Figure 2:**
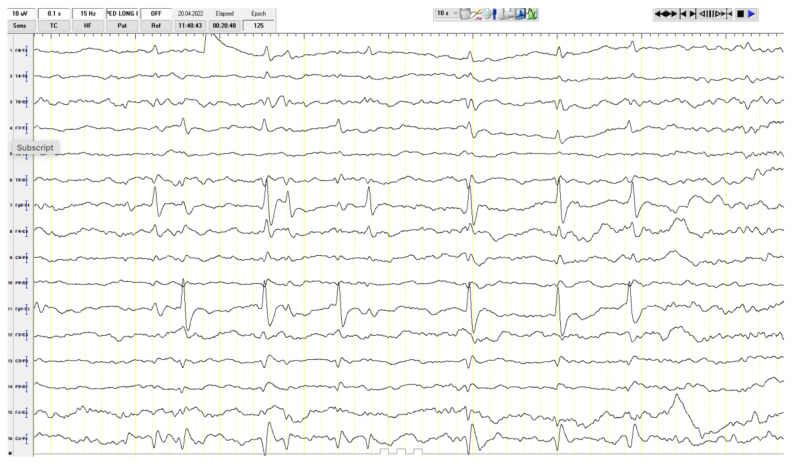
Interictal EEG recording of the patient. Representative segment of EEG, recorded during wakefulness using a longitudinal bipolar montage, showing focal high-amplitude epileptiform activity.

**Figure 3:**
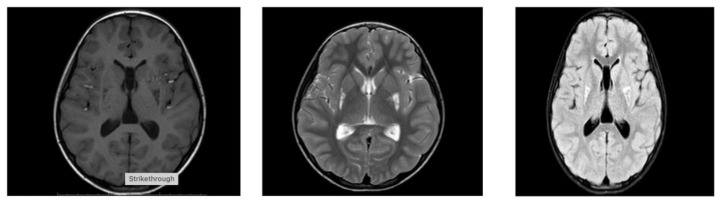
Hypointensity on T1 and hyperintensity on T2 and T2 FLAIR sections in the lower half of the bilateral lenticular nuclei.

Among the laboratory tests, her lactate level was 2.3 mmol/L (normal value is 1.5–1.63 mmol/L), while other values were within normal limits. Eye examination, hearing tests, and EMG were normal. A focal epileptiform finding was present at EEG (Figure 2). The patient’s abdominal ultrasound, along with cardiac examination, were assessed as normal. Hypointense involvement on T1 and hyperintensive involvement on T2 FLAIR images were observed in the lower half of the bilateral lenticular nuclei on cerebral MRI (Figure 3). The patient was started on mitochondrial cocktail therapy, and it was decided to continue with physical therapy.

**Figure 4:**
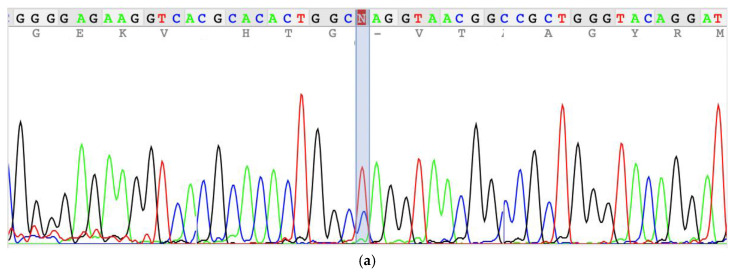

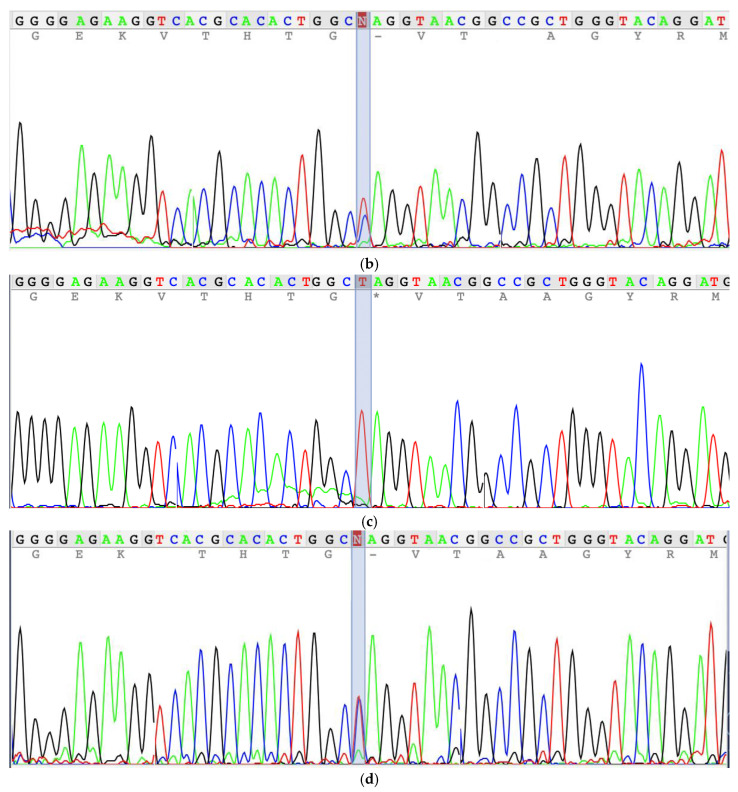
Sequencing electrogram showing the c.130C>T (p.Gln44*) variation in mother (**a**), father (**b**), index patient (**c**), and sister (**d**). The position of the variation is highlighted.

Whole-exome sequencing identified a novel homozygous nonsense variant in *NDUFS6* (NM_004553.6:c.130C>T; p.Gln44*). The variant is predicted to cause premature termination at position 44 of the 124–amino acid NADH dehydrogenase [ubiquinone] iron–sulfur protein 6, mitochondrial (NDUFS6) protein encoded by the *NDUFS6* gene. As a result, the truncated protein is expected to lack an 81–amino acid region including the zinc-finger domain, leading to a substantial impairment of protein function (NDUFS6_HUMAN, https://www.uniprot.org/uniprot/O75380, accessed on 29 January 2026). Although this variant has not been previously reported in the literature, its predicted truncating effect suggest that it is likely to be clinically significant, consistent with the pathogenicity observed for other reported *NDUFS6* variants. *NDUFS6* encodes a 13 kDa nuclear-encoded accessory subunit of mitochondrial complex I that localizes to the matrix arm and contributes to stabilization of the N-module. Functional studies with the previously published c.186+2T>A (p.Val63Glufs*9), the missense variant c.344G>A (p.Cys115Tyr), and the truncating variant c.352C>T (p.Gln118*) showed that NDUFS6 results in dissociation of most of the N-module and destabilization of complex I [1]. Consistent with this model, the variant was classified as likely pathogenic based on PVS1 (null variant in a gene with established loss-of-function disease mechanism) and PM2 (absent or very rare in public databases) according to ACMG/AMP guidelines [17]. Segregation analysis confirmed the autosomal recessive inheritance with heterozygosity in healthy parents and her sister (Figure 4).

The patient underwent comprehensive genetic analysis, including evaluation for both single-nucleotide variants (SNVs) and copy-number variants (CNVs). No additional pathogenic or likely pathogenic variants, including structural variations, were identified that could account for the observed phenotype. The absence of other candidate variants supports the conclusion that the identified *NDUFS6* variation represents the most plausible genetic cause of the patient’s clinical presentation.

## 3. Discussion

**Figure 5:**
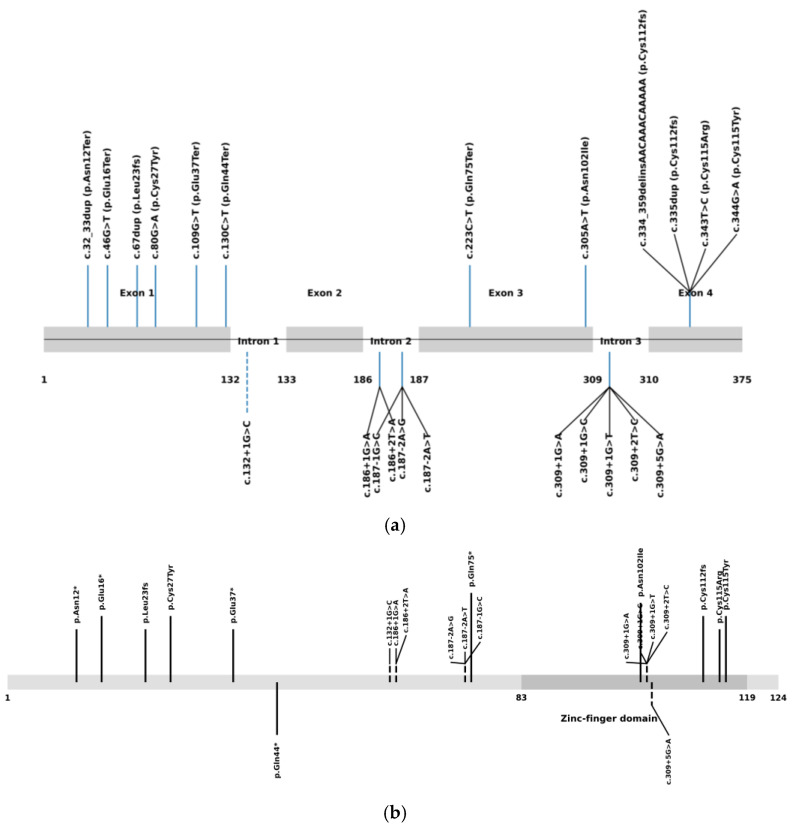
Published pathogenic *NDUFS6* variants shown at the gene (**a**) and protein (**b**) levels. The NDUFS6 protein is shown with the zinc-finger domain highlighted (amino acids 83–119). Coding variants are indicated by solid lines, while splice-site variants are shown with dashed lines and branched connectors when multiple variants affect the same splice junction. Variants associated with Leigh syndrome or Leigh-like disease are shown above the protein, whereas neuropathy/Charcot–Marie–Tooth-associated variants are shown below.

Comprehensive analysis of all published patients with biallelic *NDUFS6* variants reveals that *NDUFS6*-related disease constitutes a phenotypic continuum rather than a single clinical entity. As of December 2025, 22 pathogenic or likely pathogenic variants were reported in the Pubmed and ClinVar databases (Figure 5). Across all reported cases, biallelic *NDUFS6* variants give rise to a broad clinical spectrum ranging from neonatal lethal mitochondrial disease to childhood- or adolescent-onset axonal neuropathy/Charcot–Marie–Tooth (CMT)-like phenotypes (Table 1). Early reports predominantly described neonates presenting within the first days of life with profound hypotonia, severe lactic acidosis, and rapid clinical deterioration leading to death, often in association with truncating or frameshift variants and minimal residual complex I function. A subset of patients presented later in infancy with Leigh syndrome or Leigh-like disease, characterized by developmental regression, characteristic basal ganglia involvement on neuroimaging, elevated lactate levels, and poor outcomes [9,10,11,12,13,14,15].

In contrast, recent studies reported a distinct neuropathy-predominant phenotype, associated with the recurrent splice-region variant c.309+5G>A [7,16]. Six patients in two studies were found to carry c.309+5G>A mutation and manifest axonal sensorimotor neuropathy with pes cavus, steppage gait, and minimal disease progression, often with normal or near-normal brain MRI and only mildly elevated or normal lactate levels. Importantly, epilepsy has traditionally been associated with the encephalopathic end of the *NDUFS6* disease spectrum. However, Armirola-Ricaurte et al. [16] reported epilepsy in patient II of family 3, who predominantly exhibited a neuropathy-dominant phenotype. The presence of epilepsy in our patient provides independent confirmation that seizures can occur within *NDUFS6*-associated neuropathy. Collectively, these observations imply a phenotypic spectrum of *NDUFS6*-related disease, in which partial complex I dysfunction variably affects both the peripheral and central nervous systems.

*NDUFS6* encodes a 13 Kda nuclear-encoded accessory subunit located in the matrix arm of complex I and contributes to stabilization of the N-module. Functional studies provide a mechanistic framework to explain this variability. Experimental knockout of *NDUFS6*, as reported by Stroud et al. [1], demonstrated that complete loss of NDUFS6 leads to dissociation of most of the N-module and destabilization of mitochondrial complex I. Importantly, despite this pronounced structural defect, complex I enzymatic activity and overall respiratory capacity were not severely impaired, indicating that NDUFS6 is critical for structural stability and assembly, rather than for the catalytic function of the enzyme. Interestingly, pathogenic *NDUFS6* variants reported in patients—including the splice-site variant c.186+2T>A (p.Val63Glufs*9), the missense variant c.344G>A (p.Cys115Tyr), and the truncating variant c.352C>T (p.Gln118*)—have been clinically associated with severe neonatal phenotypes, yet remain compatible with partial complex I function.

Functional experiments were not performed in the present study due to limited experimental facilities. Nevertheless, inference regarding the functional impact of the identified variant is supported by previously published studies demonstrating that truncating *NDUFS6* variants result in loss of protein function and destabilization of mitochondrial complex I. In particular, functional analyses by Stroud et al. [1] showed that complete loss of *NDUFS6* leads to dissociation of the N-module and impaired assembly of complex I without abolishing catalytic activity, indicating a primary role in structural stability and assembly rather than enzymatic function. Similar truncating *NDUFS6* variants reported in patients with severe mitochondrial disease have also been shown to be associated with reduced protein levels and complex I instability. Accordingly, we infer that our novel homozygous *NDUFS6* nonsense variant (c.130C>T, p.Gln44*) is likewise expected to cause loss of NDUFS6 function and impaired complex I assembly.

The recurrent neuropathy-associated splice variant c.309+5G>A disrupts the canonical exon 3 donor site, leading to aberrant splicing and markedly reduced levels of the full-length transcript, while still allowing expression of partially functional isoforms that retain the critical Cys115 residue within the zinc-finger domain [16]. This residual complex I function provides a mechanistic explanation for survival beyond infancy and accounts for the emergence of later-onset, neuropathy-predominant, or Charcot–Marie–Tooth-like phenotypes, occasionally accompanied by central nervous system involvement such as epilepsy. Here, we have presented that neuropathy is not restricted to the recurrent splice-site variant c.309+5G>A. By reporting a patient with a novel homozygous truncating *NDUFS6* variant and a neuropathy-predominant phenotype, our study shows that mutations at different positions within the *NDUFS6* gene may be associated with peripheral neuropathy, further underscoring the phenotypic heterogeneity of *NDUFS6*-related disease.

Current evidence does not support a clear genotype–phenotype correlation for pathogenic *NDUFS6* variants. Both truncating and splice-site mutations in *NDUFS6* have been associated with markedly different clinical outcomes, ranging from neonatal lethal mitochondrial disease and Leigh syndrome to later-onset axonal neuropathy and Charcot–Marie–Tooth-like phenotypes. Notably, mutations predicted to cause complete loss of protein function, including nonsense, frameshift, and canonical splice-site variants, have been reported in patients with both severe encephalopathic presentations and comparatively mild neuropathy-predominant disease. Additionally, no clear genotype–phenotype correlation is observed between patients carrying homozygous or compound heterozygous *NDUFS6* mutations. This phenotypic variability is further supported by functional data from Stroud et al. [1], who demonstrated that complete knockout of *NDUFS6* leads to dissociation of the N-module and destabilization of mitochondrial complex I without severely impairing enzymatic activity or respiratory capacity. These findings indicate that NDUFS6 is essential for the structural stability and assembly of complex I rather than for its catalytic function, and that substantial residual complex I activity may persist even in the context of truncating variants. Consequently, clinical severity in *NDUFS6*-related disease is unlikely to be determined solely by mutation type and is more plausibly influenced by additional factors such as residual transcript expression, tissue-specific vulnerability, mitochondrial compensatory mechanisms, and genetic or environmental modifiers.

Our patient clearly aligns with this neuropathy-dominant spectrum. Despite carrying a truncating *NDUFS6* variant predicted to cause loss of protein function, she did not develop neonatal metabolic decompensation. Instead, she manifested a slowly progressive motor neuropathy with orthopedic complications, pyramidal signs, and mild basal ganglia involvement. The presence of urinary incontinence suggests possible autonomic involvement, further expanding the neurological manifestations associated with NDUFS6 deficiency.

## 4. Materials and Methods

Written informed consent was obtained from the parents of the patient. A comprehensive clinical history was obtained, and neurological examinations were performed for all family members. Genomic DNA was extracted from peripheral blood and whole-exome sequencing (WES) was performed by capture of the coding regions and splice sites of targeted genes using the Twist Human Core Exome (Twist Bioscience, South San Francisco, CA 94080 USA). After library enrichment and quality control, the samples were sequenced using the Illumina HiSeq4000 (Illumina, Inc. San Diego, CA 92122 USA) instrument with 100 bp paired-end reads at an average sequencing depth of 100×. An average read depth of 20× and 95% coverage, including exon–intron junction boundaries (±10 bp), were evaluated. Variant filtering considered allele frequency, predicted impact, inheritance pattern, and phenotypic relevance [18]. The reference human genome (hg19/GRCh37) was used for the analysis. Human Phenotype Ontology was used for phenotypic filters, and Online Mendelian Inheritance in Man (OMIM, https://www.omim.org/, accessed on 2 December 2025) was used for gene sets. The raw data was uploaded and analyzed on Franklin (https://franklin.genoox.com, accessed on 2 December 2025). Both single-nucleotide variants (SNVs) and copy-number variants (CNVs) were investigated. The variants were classified according to the American College of Medical Genetics and Genomics (ACMG) criteria [17]. Confirmation of the identified variant within the family was performed using Sanger sequencing on an ABI PRISM 3130 Genetic Analyzer (Applied Biosystems, Thermo Fisher Scientific, Tokyo, Japan).

Clinical evaluation included detailed neurological examination, gait assessment, strength testing, and review of autonomic symptoms. Electroencephalography (EEG), metabolic laboratory studies, electromyography (EMG), and ophthalmologic and audiologic evaluations were obtained. Brain magnetic resonance imaging (MRI) included T1-weighted, T2-weighted, and Fluid-Attenuated Inversion Recovery (FLAIR) sequences.

A comprehensive review of all published patients with *NDUFS6* variants was performed, and the main findings from the previously reported cases, alongside the present index cases, are summarized in Table 1.

## 5. Conclusions

This study identifies a novel homozygous truncating *NDUFS6* variant associated with childhood-onset axonal neuropathy accompanied by pyramidal signs, further expanding the phenotypic spectrum of *NDUFS6*-related disease. While neuropathy has previously been reported primarily in association with the recurrent splice-site variant c.309+5G>A, our findings demonstrate that truncating *NDUFS6* mutations can also underlie a neuropathy-predominant phenotype. Integration of all published cases highlights the marked clinical heterogeneity associated with NDUFS6 deficiency, encompassing presentations that range from lethal neonatal mitochondrial encephalopathy to slowly progressive hereditary neuropathy.

These observations support the inclusion of *NDUFS6* in the diagnostic evaluation of neuropathy, Charcot–Marie–Tooth-like phenotypes, and unexplained pes cavus deformity, particularly in consanguineous families or in patients with subtle or atypical mitochondrial features. Collectively, the present study provides further evidence that *NDUFS6* is a bona fide neuropathy-associated gene and justifies its inclusion in diagnostic gene panels for inherited peripheral neuropathies and Charcot–Marie–Tooth disease.

## Figures and Tables

**Table 1:** Clinical features of patients with *NDUFS6* variant in the present report and previous reports.

Reference	Variation	Phenotype	Onset	Brain MRI	Blood Lactate	Outcome
Kirby et al., 2004 [9]	deletion: chr5:g.1,865,477_1,869,744del	Neonatal lethal mitochondrial disease, profoundly hypotonic and drowsy, abnormal, slowly drifting eye movements, rolling nystagmus	Neonatal	-	12.0 mmol/L (normal < 2.5)	Died: 6 d; Central hypoventilation
Kirby et al., 2004 [9]	c.186+2T>A (p.V63Efs*9)	Neonatal lethal mitochondrial disease, profoundly hypotonic and drowsy, abnormal, slowly drifting eye movements, rolling nystagmus	Neonatal	Cerebral CT scan normal	6.4 mmol/L (normal < 2.5)	Died: 11 d; Central hypoventilation
Kirby et al., 2004 [9]	c.186+2T>A (p.V63Efs*9)	Neonatal lethal mitochondrial disease, profoundly hypotonic and drowsy	Neonatal	Cerebral CT scan normal	6.7 mmol/L (normal < 2.5)	Died: 6 d; Central hypoventilation
Spiegel et al., 2009 [10]	c.344G>A (p.Cys115Tyr)	Neonatal lethal mitochondrial disease, encephalopathy, severe lactic acidosis	2 d	-	-	Died/Outcome: 8 d
Spiegel et al., 2009 [10]	c.344G>A (p.Cys115Tyr)	Neonatal lethal mitochondrial disease, lethargic, drowsy, refused to eat	2 d	Postnatal brain ultrasound normal	6.0–11.2 mmol/L (normal < 2.0)	Died: <10 d; Severe metabolic acidosis
Spiegel et al., 2009 [10]	c.344G>A (p.Cys115Tyr)	Neonatal lethal mitochondrial disease, apathy, tachypnea, severe metabolic acidosis	6 d	-	-	Died: 6 d; Severe metabolic acidosis
Spiegel et al., 2009 [10]	c.344G>A (p.Cys115Tyr)	Neonatal lethal mitochondrial disease, pale, tachypneic, severely hypotonic, unresponsive	6 d	-	16.8 mmol/L (normal < 2.0)	Died/Outcome: 8 d
Haack et al., 2012 [11]	c.352C>T (p.Gln118*)	Other/unspecified	<6 mo	Normal	Elevated	Alive
Pronicka et al., 2016 [12]	c.313_315delAAAG and c.334_359del126ins13 (p.104Lys_106Thrfs and p.Glu112fs)	Neonatal lethal mitochondrial disease	Neonatal	Normal	-	Not reported
Ogawa et al., 2017 [13]	c.309+5G>A (p.?) and c.343T>C (p.Cys115Arg)	Leigh syndrome/Leigh-like	-	-	-	Not reported
Rouzier et al., 2019 [14]	c.309+5G>A (p.?) and c.343T>C (p.Cys115Arg)	Leigh syndrome/Leigh-like	4 mo	Consistent with Leigh syndrome	4,74 mmol/L (normal < 2.5)	Died/Outcome: 11 months
Li et al., 2022 [15]	c.344G >T (p.Cys115Phe)	Neonatal lethal mitochondrial disease	13 d	-	Elevated	Died: 27 d; Ineffective treatment
Gangfuß et al., 2024 [7]	c.309+5G>A (p.?)	Axonal neuropathy/CMT, abnormal gait with frequent falls, pronounced axonal, sensory, and motor neuropathy (reduced latency and amplitudes, mostly normal conduction velocities)	7 y	Cerebral MRI at 10 yr normal	2.5–2.8 mmol/L (normal 0.5–1.6)	Alive at 10 y
Armirola-Ricaurte et al., 2024 [16]	c.309+5G>A (p.?)	Axonal neuropathy/CMT, distal weakness/atrophy, pes cavus, steppage gait, nystagmus	1 y	Normal	1.9 mmol/L	Alive/Not reported
Armirola-Ricaurte et al., 2024 [16]	c.309+5G>A (p.?)	Axonal neuropathy/CMT, distal weakness, pes cavus, steppage gait, nystagmus, minimal progression, intellectual disability	10 y	Normal	1.03 mmol/L	Alive/Not reported
Armirola-Ricaurte et al., 2024 [16]	c.309+5G>A (p.?)	Axonal neuropathy/CMT, unsteady gait/falls, pes cavus, steppage gait, minimal progression, involuntary movements	10 y	Eye-of-the-tiger sign reported (basal ganglia)	2.02 mmol/L	Alive/Not reported
Armirola-Ricaurte et al., 2024 [16]	c.309+5G>A (p.?)	Axonal neuropathy/CMT, unsteady gait/falls, pes cavus, steppage gait, minimal progression, Rolandic epilepsy, involuntary movements	10 y	--	--	Alive/Not reported
Present study	c.130C>T (p.Gln44*)	Axonal neuropathy, childhood-onset motor-neuropathy-like phenotype with pes cavus, knee-flexion gait, impaired heel/tandem gait, tendon-release surgery, urinary incontinence, focal epileptiform EEG	3 y	Bilateral lenticular nuclei T1 hypointensity and T2/T2-FLAIR hyperintensity	2.3 mmol/L	Alive

## Data Availability

The original contributions presented in this study are included in the article. Further inquiries can be directed to the corresponding author.
